# Mycorrhizal fungi mediate the direction and strength of plant–soil feedbacks differently between arbuscular mycorrhizal and ectomycorrhizal communities

**DOI:** 10.1038/s42003-018-0201-9

**Published:** 2018-11-20

**Authors:** Kohmei Kadowaki, Satoshi Yamamoto, Hirotoshi Sato, Akifumi S. Tanabe, Amane Hidaka, Hirokazu Toju

**Affiliations:** 1 Center for Ecological Research, Kyoto University, Hirano 2, Otsu, Shiga 520-2113 Japan; 2 Research and Educational Unit for Studies on Connectivity of Hills, Humans and Oceans, Kyoto University, Kitashirakawa Oiwake-cho, Sakyo, Kyoto 606-8502 Japan; 3 Graduate School of Science, Kyoto University, Kitashirakawa Oiwake-cho, Sakyo, Kyoto 606-8502 Japan; 4 Graduate School of Human and Environmental Studies, Kyoto University, Yoshida Nihonmatsu-cho, Sakyo, Kyoto 606-8501 Japan; 5 Faculty of Science and Technology, Ryukoku University, 1-5 Yokotani, Seta Oe-cho, Otsu, Shiga 520-2194 Japan; 6 Graduate School of Agriculture, Kyoto University, Kitashirakawa Oiwake-cho, Sakyo, Kyoto 606-8502 Japan; 7 Precursory Research for Embryonic Science and Technology (PRESTO), Japan Science and Technology Agency, Kawaguchi, Saitama 332-0012 Japan

## Abstract

Plants influence their soil environment, which affects the next generation of seedlings that can be established. While research has shown that such plant–soil feedbacks occur in the presence of mycorrhizal fungi, it remains unclear when and how mycorrhizal fungi mediate the direction and strength of feedbacks in tree communities. Here we show that arbuscular mycorrhizal and ectomycorrhizal fungal guilds mediate plant–soil feedbacks differently to influence large-scale patterns such as tree species coexistence and succession. When seedlings are grown under the same mycorrhizal type forest, arbuscular mycorrhizal plant species exhibit negative or neutral feedbacks and ectomycorrhizal plant species do neutral or positive feedbacks. In contrast, positive and neutral feedbacks dominate when seedlings are grown in associations within the same versus different mycorrhizal types. Thus, ectomycorrhizal communities show more positive feedbacks than arbuscular mycorrhizal communities, potentially explaining why most temperate forests are ectomycorrhizal.

## Abstract (web summary)

Kadowaki et al. examine species-specific feedbacks that occur within mycorrhizal fungal guilds in the establishment of tree seedling-soil microbiomes. Using reciprocal invasion experiments, they show that the form of symbiotic relationship affects the nature of microbial feedback.

## Introduction

Feedbacks between plant community assembly and soil biota are critical to understanding the dynamics of forest ecosystems such as coexistence and succession^1–4^. Plant–soil feedbacks influence seedling community assembly when the effects of soil biota that reside in association with a given plant species are expressed more strongly on conspecific than on heterospecific seedlings^1,5–7^. Recent studies have emphasized that the direction and strength of plant–soil feedbacks can be explained by the mycorrhizal fungal types (or guilds) of plant species^8,9^. Negative feedbacks are generally limited to arbuscular mycorrhizal plant species, and positive feedbacks are typically observed in ectomycorrhizal plant species^8,9^. The negative feedbacks of arbuscular mycorrhizal plant species increase the abundance of soil biota that make the soil less suitable for conspecific seedlings relative to heterospecifics, thereby promoting the coexistence of different arbuscular mycorrhizal plant species at the community level^7,9–12^. In contrast, the positive feedbacks of ectomycorrhizal plant species increase the abundance of soil biota that favor conspecific seedlings over heterospecifics, thereby promoting the dominance of the ectomycorrhizal plant species within a community^8,13,14^.

While many studies have assessed the direction and strength of plant–soil feedbacks within the same mycorrhizal type (i.e., growth responses of arbuscular mycorrhizal seedlings under arbuscular mycorrhizal resident trees, or those of ectomycorrhizal seedlings under ectomycorrhizal resident trees), few studies have quantified feedbacks when resident plants and colonizing seedlings have mismatched mycorrhizal types. Bennett et al.^9^ measured feedbacks using the growth responses of seedlings in soils conditioned by conspecifics and heterospecifics. They concluded that the direction and strength of feedbacks were generally species-specific, and hence plant species identity (i.e., whether the resident species is conspecific or heterospecific) could be a more important predictor of plant–soil feedbacks than mycorrhizal type match/mismatch. Nevertheless, given that arbuscular mycorrhizal and ectomycorrhizal plants co-occur across broad climatic ranges and that ectomycorrhizal forests frequently have an arbuscular mycorrhizal understory in temperate zones^14^, matching/mismatching of mycorrhizal type between resident plants and recruited seedlings may play a prominent role in driving plant community dynamics^9,15,16^. For example, ectomycorrhizal seedlings may grow faster than arbuscular mycorrhizal seedlings when colonizing forests dominated by ectomycorrhizal trees, generating positive feedbacks as a consequence of mycorrhizal type matching. Alternatively, negative feedbacks may allow for species with contrasting mycorrhizal types to coexist in mixed communities as a result of the suppression of dominant species^5,17^. Given that trees may attempt to recruit into areas that are more homogeneously dominated by either arbuscular mycorrhizal or ectomycorrhizal plants (not mixed compositions as in Bennett et al.^9^), mycorrhizal type matching could govern the outcome of plant–soil feedbacks with possible consequences for seedling community assembly^17,18^.

To better understand how plant–soil feedbacks affect seedling community assembly in natural communities, emphasis should shift from the feedback effects/responses of one plant species on another (e.g., home-versus-away experiments) to the feedbacks in multi-species community contexts. In such contexts, plant–soil feedbacks may emerge as a general community-scale process, where multiple seedlings and residents collectively form common mycelial networks belowground via mycorrhizal fungi^19–22^. These networks have the potential to modulate how resident species modify the soil biotic properties and how seedling species respond to these changes through a broad set of mechanisms (e.g., transfers of nutrients among connected resident and seedling species^18,20,23^ and modifying biogeochemical cycling^24,25^). While there is some evidence that plant–soil feedbacks have a greater impact in mixed-species communities^10^, few studies have investigated feedbacks in the presence of microbiota potentially connecting neighboring resident trees and seedlings via mycelial networks. Such community-scale feedbacks may influence seedling community assembly differently from the commonly studied species-pairwise feedbacks (possibly through different belowground mechanisms).

Here we examine whether community-scale plant–soil feedbacks affect seedling community assembly and, if so, how these effects may be linked with mycorrhizal type match/mismatch. Using experimental mesocosms simulating mixed-species forest stands, we established resident sapling communities carrying mycorrhizal inocula (the conditioning phase) and then introduced uninoculated seedling communities into the mesocosms and followed the subsequent growth of the seedlings (the feedback phase). By implementing this experiment in a fully factorial design—that is, by varying mycorrhizal type of resident forest types (arbuscular mycorrhizal, ectomycorrhizal, and control [mimic trees]) and seedling community types (arbuscular mycorrhizal, ectomycorrhizal, and control [no seedlings])—we are able to make a direct inference regarding how mycorrhizal type match/mismatch mediates plant–soil feedbacks in the presence of soil microbiota at the community level. Specifically, by adopting a spatially hierarchical design where sapling species treatments are nested within a mesocosm, we address two-layered predictions about how seedling species respond to the soil conditions modified by conspecific/heterospecific saplings, and/or by matching/mismatching mycorrhizal types. We used seedlings as phytometers to measure the properties of neighboring saplings (i.e., conspecific versus heterospecific sapling microenvironments) and mesocosm (i.e., mycorrhizal type).

Our findings indicate that seedlings generally exhibited negative to positive feedbacks depending on sapling-seedling species combinations. When comparing plant–soil feedbacks associated with conspecific versus heterospecific saplings within mesocosms with same mycorrhizal type (i.e., arbuscular mycorrhizal seedlings grown in arbuscular mycorrhizal resident forests, or ectomycorrhizal seedlings grown with ectomycorrhizal resident forests), we tended to detect negative or neutral feedbacks for arbuscular mycorrhizal plant species and neutral or positive feedbacks for ectomycorrhizal plant species. In contrast, when comparing plant–soil feedbacks associated with heterospecific saplings in matching versus mismatching mesocosms (i.e., arbuscular mycorrhizal seedlings grown in ectomycorrhizal resident forests, and ectomycorrhizal seedlings grown in arbuscular mycorrhizal resident forests), we found positive to neutral feedbacks at the mesocosm-scale. We conclude that the assembly of a temperate tree community may be determined by a combination of species-specific plant–soil feedbacks within the same mycorrhizal fungal guild and positive plant–soil feedbacks driven by the match/mismatch of mycorrhizal type between resident plants and seedlings. By accounting for community-scale plant–soil feedbacks, we will be able to consider how tree species of the same mycorrhizal type can coexist, and why ectomycorrhizal trees, but not arbuscular mycorrhizal trees, can become dominant in late-successional temperate forest communities.

## Results

### Experimental setup

**Fig. 1:**
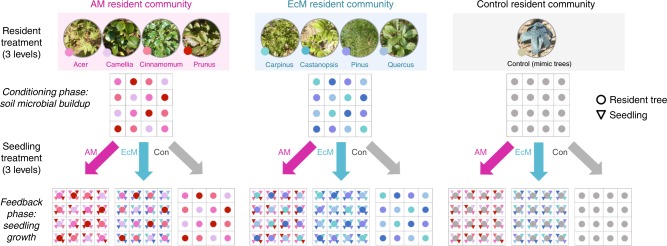
Schematic depiction of the experimental design. A two-way factorial design of the reciprocal invasion experiment, using three by three resident–seedling treatment combinations. The experiment was undertaken following two steps: the conditioning phase and the feedback phase. In the conditioning phase, we established saplings carrying mycorrhizal inocula into the mesocosms filled with soil, each community consisting of 16 saplings (represented by 4 species × 4 individuals per species, each species represented by different color). In the feedback phase, we planted 16 seedlings per species per mesocosm (i.e., four seedlings of different species (triangles) planted at four inter-cardinal positions in each grid cell) and allowed them to grow in interaction with saplings and their belowground fungal associates. For the resident control treatments, mimic trees made of poles and cheesecloth were planted, and for the seedling control treatments, no seedlings were added to the mesocosms. Note that blocks are our true unit of replication of independent treatments; phytometers (i.e., seedlings) of species per mesocosm are a form of subsampling (technical replicates or pseudoreplicates). Photos by K Kadowaki

**Fig. 2:**
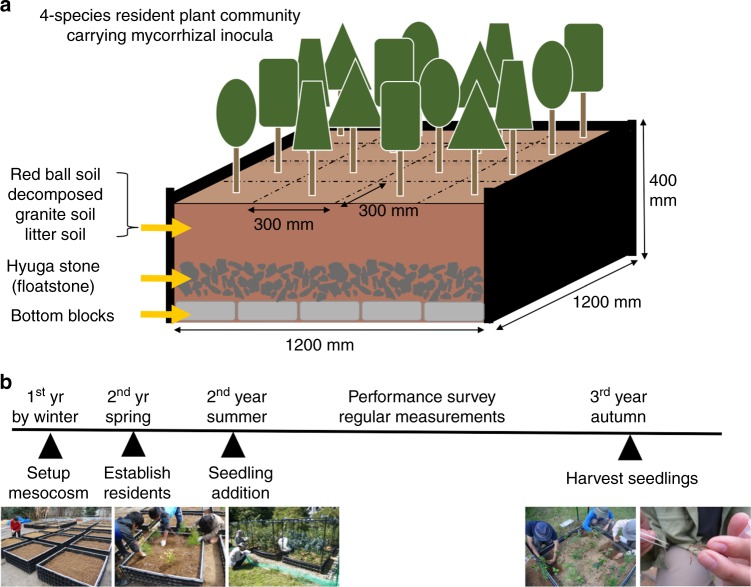
Mesocosm layout, and the timeline of research. **a** Illustration of mesocosm (1.2 m × 1.2 m × 0.4 m height). Each mesocosm (measuring 1.2 m × 1.2 m × 0.4 m height) consisted of multiple soil horizons (Fig. 2a): 12 cm layer of concrete blocks as bedrock, 72 and 36 L of Hyuga stones (large- and small-grain float-stones, respectively) that collectively ensure water drainage, 270 L of a mixture of red-ball soil (70%), decomposed granite soil (20%), and litter soil (10%). Starting from soil with low organic matter content minimized potential differences in decomposition and mineralization across replicate mesocosms. The bottom was covered by fine mesh to minimize colonization by soil animals. Red ball soil (akadama soil), the base soil component used in our study, retains water and nutrients while providing porosity and free drainage, and hence is suitable as growing medium of woody species. Each mesocosm was surrounded by a 30-cm-wide non-vegetated border (filled by decomposed granite soil) to reduce edge effects and was raised 15 cm from the border soil surface, thus allowing no exchange of soil inside/outside the mesocosms. The study site was installed in an old fallow field, rotovated, and completely leveled off. **b** Timeline represents times when major activities in the study occurred

In the community-scale experiment crossing three resident forest types (four-species arbuscular mycorrhizal sapling community, four-species ectomycorrhizal sapling community, and control [mimic trees]) and three seedling community-types (four-species arbuscular mycorrhizal community, four-species ectomycorrhizal community, and control [no seedlings]) (9 combinations ×4 block replicates = 36 mesocosms in a randomized block design), we assessed seedling growth responses under different resident conditions over two growing seasons (Figs. 1 and 2). To initiate the conditioning phase, a resident forest community was set up in each mesocosm with four resident species (four saplings each) arrayed in 4 × 4 Latin square grids in order to avoid spatial clumping of sapling species (Fig. 1b; for the list of species used, see Methods and Supplementary Table 1). Then, at the feedback phase, each seedling species was planted underneath the sapling in each grid cell, and a total of 64 seedlings (all four seedling species combined) were placed into the resident forest (Figs. 1 and 2; see also Methods). Based on this experimental design, we were able to address how seedling species responded to the soil conditions modified by conspecific/heterospecific saplings within a mesocosm, and/or mycorrhizal type (i.e., matched/mismatched) of the mesocosm. Hereafter, we focus on these layered questions, and the background information about seedling growth responses and the evidence of sapling–seedling interactions are provided in Supplementary Information (see Supplementary Figure 1 and Supplementary Note 1).

### Seedling growth responses under conspecific versus heterospecific sapling species

Using seedling biomass, *G*_*x*_(*y*) (the averaged biomass of seedling species *x* under sapling species *y* in a mesocosm; Supplementary Figure 2) as a mesocosm-level indicator of growth response, we assessed how seedlings of each species responded to the soils modified by conspecific versus heterospecific saplings within the same mycorrhizal types. To do this, we built a linear mixed-effects model using *ln*-transformed growth response of seedling species *x* as the response variable, sapling species identity *y* as a fixed predictor, and spatial block as a random predictor.

**Fig. 3:**
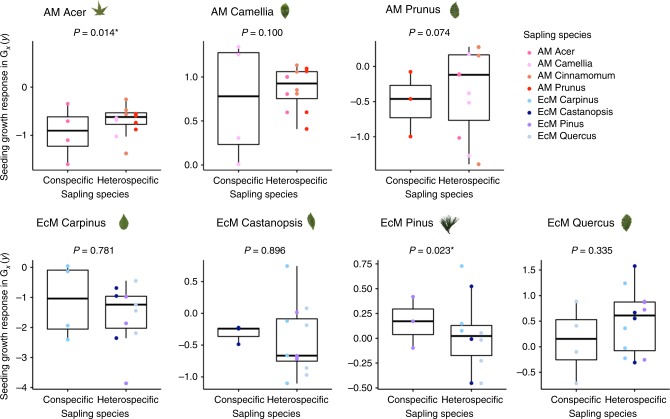
Comparison of seedling growth responses under conspecific versus heterospecific resident saplings in plant communities associated with matching mycorrhizal types. Growth response *G*_*x*_(*y*) was calculated as the average of total seedling biomass (aboveground and belowground combined) of species measured in quadruplicate per sapling species per mesocosm. Based on the fitted linear mixed-effects model, linear contrasts were made between the *ln*-transformed total biomass *G*_*x*_(*y*) of individual seedling species under conspecific versus heterospecific saplings of matching mycorrhizal types. In each seedling species, we used *ln*-transformed growth response of seedling species *x*, that is, ln[*G*_*x*_(*y*)], as the response variable, sapling species identity *y* as a fixed-effects predictor, and block as a random-effects predictor: *ln*[*G*_*x*_(*y*)] ~ sapling species + (1|block). Due to the slight modification of experimental design, the arbuscular mycorrhizal species *Celtis sinensis* occurred only as a seedling species not as a sapling species, so it was excluded from statistical analysis. The lines in each boxplot represent the minimum (whisker), lower quartile, median, upper quartile, and maximum (whisker)

The result showed that the growth responses of most seedling species were variable across different plant species. Two arbuscular mycorrhizal species *Acer palmatum* and *Prunus jamasakura* tended to exhibit lower growth in the soils conditioned by conspecific saplings relative to the soils conditioned by heterospecific saplings, indicating negative feedbacks (Fig. 3, *a priori* contrast *Z*-test, *Acer z* = –2.47, *P* = 0.014; *Prunus z* = –1.787, *P* = 0.074; for the results of linear mixed-effects models and *a priori* contrasts, see Supplementary Tables 2 and 3a). In contrast, three of the four ectomycorrhizal species displayed neutral feedbacks (i.e., no significant growth differences between conspecific and heterospecific sapling environments); a notable exception is that the ectomycorrhizal species *Pinus densiflora* showed significantly higher growth under conspecific saplings within the same mycorrhizal type, indicating positive feedbacks (*Z*-test, *z* = 2.272, *P* = 0.023). While the growth differences between conspecific versus heterospecific saplings varied depending on sapling–seedling species combinations, our results show some signature of negative or neutral feedbacks in arbuscular mycorrhizal plant species and neutral or positive feedbacks in ectomycorrhizal plant species when seedlings were grown in the soil conditioned by saplings of the same mycorrhizal types.

### Seedling growth responses under matching or mismatching mycorrhizal type

**Fig. 4:**
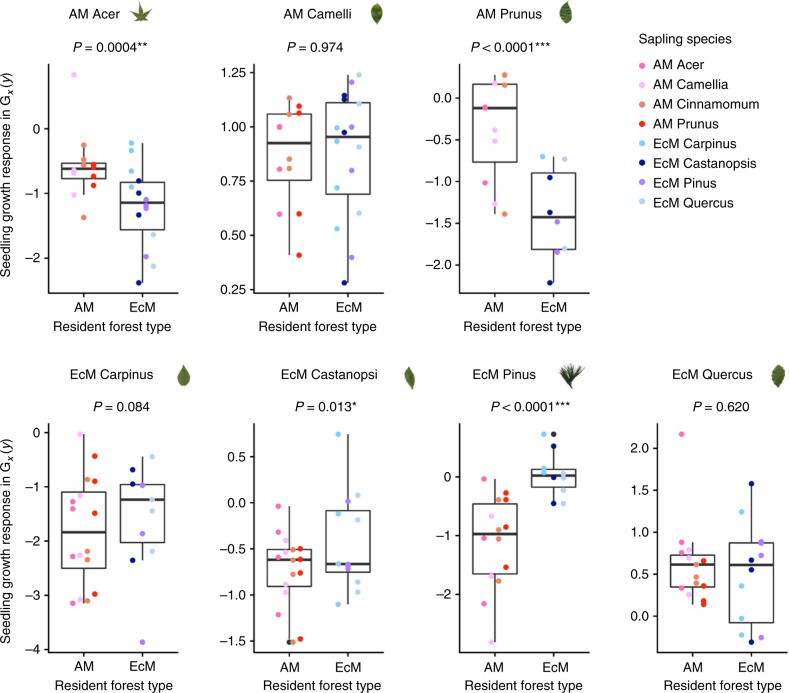
Comparison of seedling growth responses under heterospecific saplings in mesocosms associated with matching versus mismatching mycorrhizal types. Growth response *G*_*x*_(*y*) was calculated as the average of total seedling biomass (aboveground and belowground combined) of species measured in quadruplicate per sapling species per mesocosm. Based on the fitted linear mixed-effects model, linear contrasts were made between the *ln*-transformed total biomass *G*_*x*_(*y*) of individual seedling species under heterospecific saplings of matching versus mismatching mycorrhizal types. Due to the slight modification of experimental design, the arbuscular mycorrhizal species *Celtis sinensis* occurred only as a seedling species not as a sapling species, so it was excluded from statistical analysis. The lines in each boxplot represent the minimum (whisker), lower quartile, median, upper quartile, and maximum (whisker)

Using the same model, we compared the seedling growth responses under the matching versus mismatching heterospecific saplings (i.e., excluding seedlings grown with conspecifics from the analysis). Four of eight species exhibited significantly greater growth under the matching resident saplings: the arbuscular mycorrhizal species *A. palmatum* and *P. jamasakura* showed growth increases of 69.3% and 172.0%, respectively (Fig. 4; see Supplementary Table 3b for *a priori* contrasts *Z-test*, *z* = 3.514*, P* = 4.42e−04 and *z* = 4.404*, P* = 1.06e−05, respectively), and the ectomycorrhizal species *P. densiflora* and *Castanopsis cuspidata* showed growth increases of 170.0% and 45.0% (*a priori* contrasts *z* = 5.462, *P* = 4.70e−08 and *z* = 2.482, *P* = 0.013). Such a growth-enhancing effect of mycorrhizal type matching was detected for ectomycorrhizal species *Carpinus laxiflora*, but it was not statistically significant. In contrast, the arbuscular mycorrhizal species *Camellia japonica* and ectomycorrhizal species *Quercus serrata* did not differ in their growth responses between matching and mismatching conditions. We also analyzed growth responses using leaf weight and root weight separately, and confirmed that the results were qualitatively similar as found for total seedling biomass (Supplementary Figure 3). Thus, seedling performance near heterospecific saplings in matching versus mismatching fungal mesocosms indicated positive to neutral feedbacks at the mesocosm-scale (Fig. 4).

### Sharing of mycorrhizal fungi

**Fig. 5:**
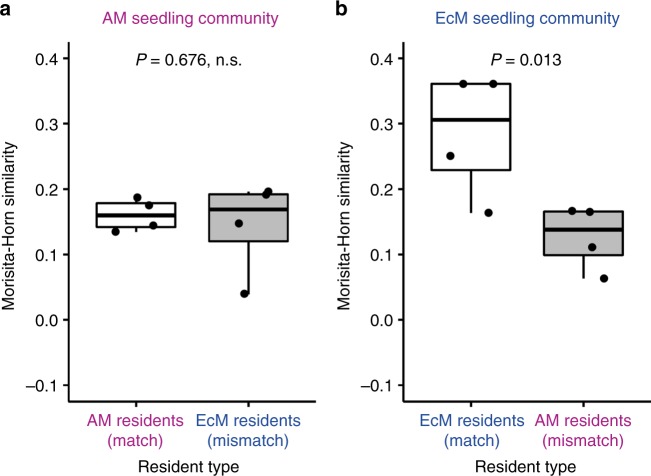
Mycorrhizal type match/mismatch affects the compositional similarities in fungal community between seedlings and saplings. The Morisita–Horn similarity index measures the extent to which seedling species shared fungal species (OTUs) with sapling species at the time of harvest. Boxplots of the Morisita–Horn similarity index are shown for matching (white) versus mismatching (gray) resident conditions (*N* = 4) for **a** arbuscular mycorrhizal (AM) seedling community and (**b**) ectomycorrhizal (EcM) seedling community. Lines in each boxplot represent the minimum (whisker), lower quartile, median, upper quartile, and maximum (whisker)

Feedbacks could become more positive when previously conditioned soil fungal biota of the matching resident forest community improved seedling growth relative to that conditioned by the mismatching forest community. To explore the possibility of the root-associated fungal community as a driver of the observed plant–soil feedbacks, we examined whether seedlings are more strongly associated with fungi associated with matching sapling communities (i.e., arbuscular mycorrhizal resident/arbuscular mycorrhizal seedling and ectomycorrhizal resident/ectomycorrhizal seedling conditions) than with mismatching sapling communities. A high-throughput DNA sequencing analysis supported this hypothesis, showing greater compositional similarity in the root-associated fungal communities of saplings and introduced seedlings under the matching condition (*F*_1,12_ = 9.002, *P* = 0.011; for an overview of the results of the sequencing analysis, see Supplementary Note 2 and Supplementary Figures 4–6). Notably, the effects of mycorrhizal type matching differed between arbuscular mycorrhizal and ectomycorrhizal symbioses (Fig. 5). For the arbuscular mycorrhizal seedling community, matching and mismatching treatments showed a similar level of fungal compositional similarity (Welch’s two-sample *t*-test, *t* = 0.452, df = 3.706, *P* = 0.676), indicating no preferential association with either resident forest type. For the ectomycorrhizal seedling community, however, matching the mycorrhizal type increased the similarity coefficient by 16.7% relative to mismatching conditions (*t* = 3.489, df = 5.923, *P* = 0.013). Thus, ectomycorrhizal seedlings were more strongly associated with fungi associated with matching resident saplings than with those associated with mismatching saplings; however, there was no such pattern for arbuscular mycorrhizal seedlings.

### Spatial structuring of plant–fungus associations

**Fig. 6:**
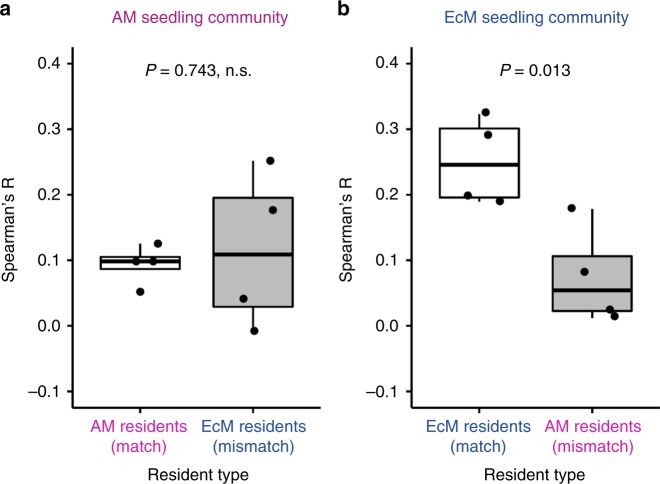
Mycorrhizal type match/mismatch shapes the spatial structuring in fungal networks connecting saplings and seedlings. As a proxy of spatial structuring of common mycelial networks, Spearman’s correlation coefficient *R* (ranging from –1 to +1) was estimated between sapling–seedling fungal community dissimilarity and physical distance (separation) within a mesocosm. A high correlation coefficient indicates that closely spaced sapling–seedling pairs within mesocosms tend to host less dissimilar fungal communities. Boxplots of Spearman’s correlation coefficients are shown for **a** arbuscular mycorrhizal (AM) seedling communities and **b** ectomycorrhizal (EcM) seedling communities under matching (white) versus mismatching (gray) conditions (*N* = 4). Lines in each boxplot represent the minimum (whisker), lower quartile, median, upper quartile, and maximum (whisker)

Considering that common mycorrhizal networks connecting seedlings and saplings might function as a community-scale driver of plant–soil feedbacks^12–14^, we tested whether more developed mycorrhizal networks with a matching resident forest community (i.e., arbuscular mycorrhizal resident/arbuscular mycorrhizal seedling and ectomycorrhizal resident/ectomycorrhizal seedling conditions) improves seedling growth relative to those with a mismatching forest community. As a proxy of the extent of development of mycorrhizal networks, we examined whether closely located sapling–seedling pairs have more similar fungal communities than more distant pairs within a mesocosm, and how the pattern of compositional similarity is spatially structured within each mesocosm. Our grid-based experimental design (Fig. 1) allowed us to evaluate such spatial patterns. Arbuscular mycorrhizal seedling communities showed weaker spatial structuring (Fig. 6), which did not depend on match/mismatch with the resident forest types (Welch’s two-sample *t*-test, *t* = –0.356, df = 3.388, *P* = 0.743). In contrast, ectomycorrhizal seedling communities displayed significantly different degrees of spatial structuring between matching and mismatching conditions (*t* = 3.489, df = 5.923, *P* = 0.013). Altogether, the ectomycorrhizal plant community developed spatially structured networks with the fungi associated with matching resident saplings, whereas the arbuscular mycorrhizal plant community did not exhibit such a pattern. Thus, the effect of soil conditioning on common mycorrhizal networks was greater for ectomycorrhizal species than for arbuscular mycorrhizal species. The results suggest that greater differentiation of the fungal communities between matching versus mismatching conditions might correspond with plant–soil feedbacks, such that ectomycorrhizal trees exert greater changes to the soil fungal community environment, which may correspond with their greater tendency for positive feedbacks.

## Discussion

Based on a mesocosm experiment of arbuscular mycorrhizal and ectomycorrhizal artificial plant communities, we examined how mycorrhizal types determine plant–soil microbiota feedbacks. Previous studies have generally reported negative feedbacks in arbuscular mycorrhizal plant species and positive feedbacks in ectomycorrhizal species^1,8,9^, and Bennett et al.^9^ emphasized that species-specific feedbacks could play a more important role than mycorrhizal type match/mismatch. We found that, in a multi-species community context, the direction and strength of feedbacks depend critically on mycorrhizal type match/mismatch. When seedlings colonize forests dominated by the matching mycorrhizal type, arbuscular mycorrhizal plant species tend to exhibit negative or neutral feedbacks and ectomycorrhizal plant species do neutral or positive feedbacks (Fig. 3). In contrast, when seedlings colonize forests dominated by the matching versus mismatching mycorrhizal type, both arbuscular mycorrhizal and ectomycorrhizal species exhibit neutral or positive feedbacks as a consequence of mycorrhizal type matching (Fig. 4). Our results suggest, when these within- and across-mycorrhizal type feedbacks occur simultaneously in natural forest, ectomycorrhizal plant species may show more positive feedbacks than arbuscular mycorrhizal plant species do. Consequently, the assembly of a temperate tree seedling community may be shaped by a combination of variable feedbacks within the same mycorrhizal guilds and positive feedbacks across different mycorrhizal guilds.

This study also revealed that the root-associated fungal community shared between saplings and seedlings may be associated with the observed patterns of plant–soil feedbacks. Specifically, for the ectomycorrhizal plant community, seedlings’ fungal symbiont acquisition and the spatial structuring of belowground fungal communities may account for the pattern that ectomycorrhizal seedlings generally performed better under the matching resident forests than under the mismatching forests (Figs. 5 and 6). We propose that the observed effects of mycorrhizal type matching on resident–seedling feedbacks may have resulted from four non-mutually exclusive mechanisms: first, more spatially extended and temporally prolonged infection of seedling roots by matching fungal communities than by mismatching ones; second, detrimental effects of incompatible mycorrhizal fungi for seedlings when grown with mismatched saplings; third, access to a larger soil nutrient pool made available by compatible fungal networks than by incompatible networks^19–21^; and fourth, more active transport of nutrients from resource-rich regions of mycelial networks to resource-poor areas^22^ via more structured hyphal networks provided by matching symbioses. The analyses of fungal communities within the mesocosms (Figs. 5 and 6) are consistent with all these possibilities. Nevertheless, the use of the internal transcribed spacer (ITS) region in the molecular analysis might have resulted in a low detection rate of arbuscular mycorrhizal fungi (see ref. ^26^ for more), and it is likely that arbuscular mycorrhizal fungal communities would have displayed spatial structuring when analyzed using DNA markers specific to arbuscular mycorrhizal fungi. While our results suggest the roles of mycorrhizal fungal communities and their belowground networks as a potential driver of plant–soil feedbacks, detailed mechanisms underlying the link between fungal symbiont acquisition and plant−soil feedbacks require further investigation.

Our approach to determining plant–soil feedbacks is different from previous research in two important ways. First, previous studies either focused on the effects of live soil inocula associated with a single resident plant species on conspecific or heterospecific seedlings (i.e., plant competition-free conditions^9,11,27^) or on the effects of one resident plant species on another in the presence of resident-seedling competition^20,28–30^. Our study assembled plant−soil communities on identical substrates of a set area, naturally and in situ at a field site, and for a known period of time, and then measured seedling growth responses under conditions in which both plant competition and mycorrhizal networks were allowed to develop. These features made it possible to examine feedbacks in more realistic, multi-species plant communities. Second, most field studies have used fungicide to test for the potential effects of soil pathogens^2,3,7,9^, but such chemical applications were necessarily confounded with possible reductions of soil mycorrhizal fungi, which could also act as key agents of feedbacks^18,25^. By combining a factorial experiment with follow-up sequencing, we were able to quantify the similarities in root-associated fungal community composition between resident trees and seedlings as a potential key agent of plant−soil feedbacks. Our findings show that the biological effect of plant−soil interactions can be placed into a broader community context, whereas it has typically been only observed in pairwise interactions.

It is necessary to note, however, some caveats of our study. First, there are some methodological limitations in our soil handling, which might potentially bias the estimation of feedbacks (see Methods for mode detail). Second, at the conditioning phase of the experiment, we chose to use mycorrhizal saplings (collected from natural forests by a local nursery) over using cultured mycorrhizal inocula. Therefore, it is possible that diverse soil biota other than mycorrhizal fungi might have driven the observed feedbacks. For instance, bacteria, soil micro-arthropods, and nematodes are also known as potential key agents of plant−soil feedbacks^2,4,10,31^, so whether they could explain the direction and strength of feedbacks observed in our study is still an open question. Third, we did not account for variation in plant species composition and richness in the mesocosm designs. For the sake of experimental feasibility and tractability, we assembled two artificial tree communities from each type to test for matching/mismatching feedbacks. If we are to confirm the generality of the findings, further studies must be undertaken to assess how the direction and strength of feedbacks differ depending on the compositions of the arbuscular mycorrhizal and ectomycorrhizal plant species used to build the experimental mesocosms. This is important because research has shown that plant–soil feedbacks can not only affect plant community structure^4,7,10^ (but see ref. ^29^) but also be affected by plant community structure^32,33^. Fourth, seedlings used in this study might have exceeded the stage susceptible to soil pathogens which might have caused underestimation of negative soil biota effects^34^, and this possibility cannot be ruled out given the scarcity of pathogens detected in our study (Supplementary Figure 4). A related issue is that such pathogens are known to deactivate under high light intensity, potentially reducing the efficacy of pathogen-mediated negative feedbacks. Despite our efforts to simulate the natural forest environment by controlling for light availability experienced by seedlings (see Experimental design described in Methods), our field experiment might have been performed in environments with higher light intensity compared to previous studies. Previous experiments and ours may thus differ in several aspects potentially influencing the functioning of plant–soil microbiota feedbacks (e.g., light intensity, soil fertility^24,35–37^), and hence the results of feedbacks should be interpreted with caution (see Supplementary Table 5 for the soil chemical profiles for our experiment).

Understanding plant–soil feedbacks in mixed-species communities and evaluating how contrasting plant mycorrhizal types shape plant−soil feedbacks are critical to predicting plant community dynamics and succession. Our findings show that the effects of plant–soil feedbacks on seedling community assembly can be modulated by mycorrhizal type match/mismatch, and such matching effects may emerge as a community-scale process in which the networks of interactions formed by soil microbiota influence the outcome of seedling community assembly. Plant–soil feedback theory predicts that stronger negative feedbacks are more likely to stabilize species coexistence within the same mycorrhizal fungal guilds (i.e., within an arbuscular mycorrhizal plant community or an ectomycorrhizal plant community), whereas more positive feedbacks observed across different mycorrhizal types are more likely to allow specific mycorrhizal plant guilds to become dominant in a forest (in our case, arbuscular mycorrhizal plant dominated forest or ectomycorrhizal plant dominated forest). Our findings may provide clues to simultaneously explain why different tree species of the same mycorrhizal type can coexist in a natural temperate forest, and why ectomycorrhizal plant communities (but not arbuscular mycorrhizal plant communities) often dominate in late-successional temperate forest.

Although the idea that mycorrhizal type is a significant predictor of plant community succession is not new^8,38^, it has not been tested experimentally at the community level. This study is a first step toward contrasting arbuscular mycorrhizal and ectomycorrhizal plant–soil feedbacks in a multi-species context and highlights the importance of simultaneously examining arbuscular mycorrhizal and ectomycorrhizal plant communities. As mycorrhizal types have been linked to plant nutritional acquisition strategies, soil properties, and nutrient cycling^24,25^, they provide a useful approach for an understanding of feedbacks at the ecosystem level. To develop a more comprehensive understanding of plant community assembly, future studies need to quantitatively evaluate the roles of both arbuscular mycorrhizal and ectomycorrhizal fungi^15,38,39^ as well as the diversity and biomass of mycorrhizal, endophytic, and pathogenic fungi in plant root systems^16,40^ in association with various abiotic factors^41^. Incorporating such complexities of real belowground plant–soil interactions will be an avenue for better predicting plant community dynamics.

## Methods

### Model system

Using plant species common to warm-temperate forests in Japan, we studied two types of model communities: a four-species arbuscular mycorrhizal plant community composed of *A. palmatum* Thunb., *C. japonica* L., *Cinnamomum camphora* (L.) J. Presl, and *P. jamasakura* (Siebold ex Koidz.) H. Ohba and a four-species ectomycorrhizal plant community composed of *Castanopsis cuspidata* (Thunb.) Schottky, *Carpinus laxiflora* (Sieb. et Zucc.) Blume, *P. densiflora* Sieb. & Zucc., and *Q. serrata* Murray (Fig. 1; for information of ecological traits of individual species, see Supplementary Table 1). The arbuscular mycorrhizal and ectomycorrhizal plant species used in this study occur sympatrically or parapatrically in secondary forests around Kyoto city, in which the experimental mesocosms of the model communities were constructed (see below).

For the conditioning phase described below (Experimental design), saplings of the eight species (≤30 cm stem height), whose roots had been infected by naturally occurring mycorrhizal fungi, were acquired from native stands by a local nursery (Nakanishi-shiseien Co. Ltd., Kyoto, Japan). Because conspecific saplings (prospective resident trees) arrived at our field station with potentially different soil fungal communities, we performed conspecific homogenization before transplanting them to the mesocosms. To do this, saplings of each species were incubated independently for 3 months using pots (60 cm × 40 cm × 20 cm) filled with non-mycorrhizal red-ball soil (akadama) in a glasshouse prior to use in the experiment. Red ball soil, the base soil component used in the conspecific homogenization step and seedling preparation (below), was surface-mined, pasteurized, and bagged by a supplier. During the pot treatment, each sapling’s root system was covered by nylon mesh, allowing mycelia (but not fine roots) to spread across to neighboring conspecific saplings within the pots. Tree saplings were then transplanted (with nylon mesh removed) to the mesocosms.

While our attempt at the conspecific homogenization step was to homogenize the microbiota of subsets of field-conditioned saplings (i.e., all conspecific resident trees), it should be noted that, following recent reviews of best practices for plant−soil feedback studies^42,43^, we recognize a potential limitation in our experimental design. Plant−soil feedback studies should avoid homogenizing soil samples/biota across experimental units (e.g., sapling species) because such methods are likely to generate potentially biased inferences^4^. Due to this reason, future mesocosm studies should either homogenize the microbiota of all resident tree species prior to the mesocosm conditioning phase or avoid homogenization steps entirely and allow for two conditioning phases (i.e., field and mesocosm)^43^. Thus, it is important to note that the soil handling methods should be selected based on research questions and goals^44^.

For the feedback phase described below (Experimental design), seeds were purchased from the nursery and individually sown. Seeds were bought from Nakanishi-shiseien Co. Ltd. and kept with moistened sphagnum moss in the refrigerator until sowing. Seeds were individually sown in 9m^3^ pots filled with non-mycorrhizal red-ball soil. The pots were maintained in a chamber covered with black plastic film (>60% shading) at 16 °C for 3 months in order to ensure that all species were of comparable size (5–10 cm stem height) at the introduction of the seedlings into mesocosms. While the red ball soil was pasteurized by a supplier before use, we could not entirely exclude the possibility that the propagated seedlings started off with mycorrhizal fungal contamination in this background soil. We planted those uninoculated seedlings directly into mesocosms (instead of sowing seeds) in order to focus on sapling effects on the seedlings that survived, not on mortality before the development of mycorrhizal symbiosis. Three-month-old seedlings should be less susceptible than germinants to various environmental stresses (e.g., heat, frost, and desiccation) and were therefore suitable for our purpose.

### Experimental design

The common garden experiment was performed in an open field established at Kyoto University Botanical Garden, Kyoto city, Japan (35°01′49″N, 135°47′10″E). We designed a full factorial mesocosm experiment involving arbuscular mycorrhizal and ectomycorrhizal plant systems to assess and compare the effects of mycorrhizal type matching on the outcome of plant–soil feedbacks. A total of 36 mesocosms were set up in four spatial blocks (each 4 m × 4 m), with each block containing nine mesocosms. The treatment design included 3 resident forest types (arbuscular mycorrhizal, ectomycorrhizal, and control [mimic trees]) × 3 seedling community types (arbuscular mycorrhizal, ectomycorrhizal, and control [no seedlings]) × 4 replicates in a randomized block design (Fig. 1).

In February 2012, we established the 36 mesocosms, each measuring 1.2 m × 1.2 m × 0.4 m, and filled them with a standardized mixture of non-sterilized red ball soil (70% of the volume), decomposed granite soil (20%), and litter soil (10%) (Fig. 2a). For arbuscular mycorrhizal and ectomycorrhizal forest type treatments, mycorrhizal saplings were initially planted to establish whole fungal communities as conditioning live inoculum in the mesocosms (*Model system*). Each mesocosm was composed of 16 saplings arrayed in a 4 × 4 Latin square (Fig. 1), with the constraint that all treatment plant species must be equally represented in every row and column within a mesocosm. Therefore, we laid a grid system to avoid spatial clumping of certain plant species within a mesocosm. During the course of the study, five *Castanopsis* saplings already established in the mesocosms were discovered to be a different ectomycorrhizal species, *Quercus glauca* (because those two species’ morphologies are very similar when small), so the data for the grid cells to which this incorrect (albeit effectively ectomycorrhizal) species were applied were removed from downstream statistical analysis.

At the feedback phase (i.e., starting 3 months after establishing resident forests; Fig. 2b), arbuscular mycorrhizal and ectomycorrhizal seedling treatments were applied to the mesocosms following a fully crossed factorial design, with introduction of the uninfected arbuscular mycorrhizal seedling community (4 seedling species × 16 individuals) and introduction of the uninfected ectomycorrhizal seedling community (4 seedling species × 16 individuals). For seedling addition treatments, seedlings (one individual for each of four seedling species) were planted directly into each of the 16 grid cells (i.e., under the 16 saplings) within a mesocosm; in each grid cell, four seedlings were planted in the four inter-cardinal positions 5 cm from each of the centered saplings, with seedling species’ positions assigned randomly in each grid cell (Fig. 1). By implementing this configuration, we were able to quadruplicate all possible species-pairwise sapling–seedling interactions (within grid cells), thereby averaging sapling–seedling interactions at the whole mesocosm scale. In the first week of the experiment, seedlings that died were replaced.

As controls, we set up three types of control mesocosms: first, no saplings were present and instead mimic trees were planted (i.e., resident control); second, no seedlings were added (i.e., seedling control); and third, neither saplings nor seedlings were present (i.e., true control). Specifically, the resident control treatment comprised 16 handmade mimic trees (made of a pole, wire, and cheesecloth), with the aim of eliminating differences across treatments in the light conditions experienced by seedlings during the feedback phase (Fig. 1). With this control treatment, we were able to focus on sapling effects via alteration of nutrient competition and plant–soil feedbacks, rather than those via changes in light availability.

After the seedling addition, we watered mesocosms every day for the first 2 weeks, and then once every 3 days in summer, and once every week in other seasons, depending on the weather, until 1 month before harvesting in 2013. We allowed for litter and spore deposition within individual mesocosms during the study. Weeds were removed on a weekly basis as soon as discovered. Individual mesocosms were covered by black shade nets that reduced sunlight by 60% during summer, and by frost-prevention nets (1.2 m high, tacked by clothes pins, 20% shading, Urin Factory Inc., Kyoto, Japan) during winter. The shading was undertaken to better simulate natural forest environment in an open field site. Throughout the experimental period, our common garden was unaffected by any detectable disturbance (*e.g*., typhoons).

We made two modifications to the experimental design, because of low germination rates of two arbuscular mycorrhizal species (*C. camphora* and *P. jamasakura*). First, *C. camphora* was replaced by a surrogate arbuscular mycorrhizal species, *Celtis sinensis* Pers., for seedling treatment; hence this species occurred only as a seedling species, not as a resident forest species. Second, we transplanted *P. jamasakura* seedlings only to the second and third columns in each mesocosm (Fig. 1).

### Harvest

Over the two growing seasons, survival, height, stem width, number of leaves, and light intensity were followed for each seedling on a quarterly basis until the final census in September 2013. At harvest, all saplings and seedlings were excavated with utmost care to separate the roots of individual plants with their aboveground biomass still attached to aid identification, especially when saplings and seedling roots intermingled. Each sample was thoroughly washed in a separate bucket filled with running tap water. For molecular analysis, we collected 2-cm pieces of about eight terminal roots from each washed sample (from all of the surviving seedlings and saplings) and stored them in 99% ethanol at –20 °C. All the raw sapling and seedling samples were placed individually in plastic bags and stored in the refrigerator until further processing for scanning, weighing dry plant samples (e.g., leaf, stem, root), and physiochemistry analysis. As a whole, we collected data for relative growth rate of seedling height; dry leaf, stem, and root weights (at harvest); total leaf area; seedling stem diameter at base; and leaf chlorophyll. We analyzed the results for total seedling biomass (dry leaf, stem, and root weights combined), and the results using leaf weight and root weight measurements separately are presented in Supplementary Figure 3. All the measurements obtained during the study are included in Supplementary Data 1.

### Fungal community structure

For each sapling and seedling sample, the composition of root-associated fungi was examined using pyrosequencing (for numbers of samples used for the analysis, see Supplementary Table 4). Each sample represented five randomly selected terminal roots (ca. 2 cm each) and was processed using the protocol detailed in the Supplementary Information (Supplementary Note 2, Next-generation sequencing analysis of root-associated fungal communities). Total DNA was extracted from each sample using the cetyltrimethylammonium bromide method. We amplified the entire ITS region and the partial ribosomal large subunit region using the fungus-specific high-coverage primer ITS1F_KYO2 and the universal primer LR3^45^. PCR was conducted with a temperature profile of 95 °C for 10 min, followed by 20 cycles at 94 °C for 20 s, 50 °C for 30 s, and 72 °C for 120 s, and a final extension at 72 °C for 7 min using the buffer and polymerase system of Ampdirect Plus (Shimadzu, Kyoto, Japan). We subjected the PCR product from each root sample to a second PCR step that targeted the ITS2 region. The second PCR was conducted using the universal primer ITS3_KYO2 fused with 454 Adaptor A and sample-specific molecular ID; the reverse universal primer was LR_KYO1b fused with 454 Adaptor B. A buffer system of Taq DNA polymerase together with standard Taq buffer (New England BioLabs, Ipswich, MA, USA) was used with a temperature profile of 95 °C for 1 min, followed by 40 cycles at 94 °C for 20 s, 50 °C for 30 s, and 72 °C for 60 s, and a final extension at 72 °C for 7 min. Sequencing was performed using a 454 GS Junior (Roche Diagnostics, Indianapolis, IN, USA).

We then performed bioinformatics analysis using Claident v0.2.2014.10.29^46^ as detailed in the Supplementary Information. Briefly, fungal operational taxonomic units (OTUs) were defined at a cutoff sequence similarity of 97%, and potentially chimeric OTUs were removed using UCHIME v4.2.40^47^. The taxonomic assignment was performed with the Query-centric auto-k-nearest neighbor method^46^ using the nt database downloaded from the NCBI ftp server (http://www.ncbi.nlm.nih.gov/Ftp/). The sequencing result table used for statistical analysis is included in Supplementary Data 2.

### Seedling growth response

We defined *G*_*x*_(*y*) as the averaged total biomass of seedling species *x* under resident sapling species *y* in a mesocosm, and used this as a mesocosm-level indicator of seedling growth response for downstream analyses (for illustration of the calculation, see Supplementary Figure 2). For example, in an ectomycorrhizal seedling × ectomycorrhizal resident mesocosm, *G*_*Pinus*_(*Carpinus*) was calculated by averaging the growth responses of *Pinus* seedlings measured in quadruplicate (per mesocosm) around the four *Carpinus* saplings (Fig. 1), and *G*_*Pinus*_(*Castanopsis*), *G*_*Pinus*_(*Pinus*), and *G*_*Pinus*_(*Quercus*) were computed as well in the same mesocosm. This calculation was repeated for all possible pairs of seedling species *x* and sapling species *y* in each mesocosm for each of the four spatial blocks (see Experimental design).

To assess the two layered predictions addressed above for individual seedling species, we used the approach of subsetting data by seedling species and fitting a linear mixed-effects model (‘lmer’ function) on these single-seedling-species datasets. In each analysis, we used *ln*-transformed growth response of seedling species *x*, that is, *ln*[*G*_*x*_(*y*)], as the response variable, sapling species identity *y* as a fixed-effects predictor, and block as a random-effects predictor. By using conspecific sapling species as the baseline, the estimated coefficient for the sapling species term takes the form of ln[*growth with heterospecific sapling G*_*x*_(*y*) */growth with conspecific sapling G*_*x*_(*x*)]), and its deviation from zero (i.e., signature of positive or negative feedbacks) can be tested using standard statistical models^44,45^. Specifically, we performed analysis using the ‘lmer’ model formula: ln[*G*_*x*_(*y*)] ~ sapling species + (1|block).

We used two forms of a priori linear contrasts to statistically assess the two predictions for each species: that seedlings grown with conspecific saplings perform better or worse than seedlings grown with heterospecific saplings and that seedlings show a greater growth response in the presence of matching relative to mismatching mycorrhizal type. We used a custom *a priori* contrast^45^ (ghlt function) to address these questions. Note that, in this model, the tests from different seedling species of the same mycorrhizal type (i.e., under matching scenarios) might not be independent from each other due to spatial associations within mesocosms. As such, the test of growth responses under saplings of the matching mycorrhizal types may provide a weaker test than that under saplings of the mismatching mycorrhizal type.

### Sharing of mycorrhizal fungi

Using molecular analysis of root-associated fungal communities (see Supplementary Figures 4, 5 and Supplementary Note 2 for the overview of the sequencing results), we examined whether seedlings exhibited greater compositional similarity of root-associated fungi with resident saplings in matching treatments (i.e., arbuscular mycorrhizal resident/arbuscular mycorrhizal seedling and ectomycorrhizal resident/ectomycorrhizal seedling) than in mismatching treatments. In so doing, we calculated the Morisita–Horn similarity coefficient (1 – *C*_MH_) in fungal OTU composition for paired sapling and seedling species (i.e., co-occurring within the same grids), and averaged the similarity coefficient across all possible sapling–seedling species pairs to obtain a mesocosm-level indicator of seedlings’ fungal symbiont acquisition (*N* = 4; for results of sapling–seedling species-pairwise similarity, see Supplementary Figure 6). Because of the slightly different spatial design between arbuscular mycorrhizal and ectomycorrhizal seedling addition treatments (see *Experimental design*), we examined differences in the mesocosm-level fungal compositional similarity between matching and mismatching treatments (each *N* = 4) for arbuscular mycorrhizal and ectomycorrhizal seedling type separately, using Welch’s *t-*test (instead of a linear model and two-way ANOVA).

### Spatial structuring of plant–fungus associations

To evaluate the tendency of seedlings to share fungal species (OTUs) with closer saplings than with distant ones, we calculated compositional dissimilarity using *C*_MH_ for every possible individual sapling–seedling pair in each mesocosm. We generated two *i* × *j* distance matrices **A** (each entry representing the dissimilarity coefficient between sapling and seedling in a mesocosm) and **B** (each entry representing physical Euclidean distance between the paired sapling and seedling):

$${\mathbf{A}} = \left[ {\begin{array}{*{20}{c}} {a_{11}} & \ldots & {a_{1j}} \\ \vdots & \ddots & \vdots \\ {a_{i1}} & \cdots & {a_{ij}} \end{array}} \right],\,{\mathbf{B}} = \left[ {\begin{array}{*{20}{c}} {b_{11}} & \ldots & {b_{1j}} \\ \vdots & \ddots & \vdots \\ {b_{i1}} & \cdots & {b_{ij}} \end{array}} \right].$$

By stacking each of the two matrices into a single column (i.e., **A** = ([*a*_11_, *a*_12_, ….*a*_*ij*_]^T^, **B** = [*b*_11_, *b*_12_, ….,*b*_*ij*_]^T^) and calculating their correlation using Spearman’s correlation coefficient, we obtained a mesocosm-level indicator *R* of belowground plant–fungus spatial structuring (*N* = 4). A higher *R* indicates that closely located sapling–seedling pairs shared a higher proportion of the fungal communities than did distantly located pairs. We examined differences in Spearman’s correlation coefficient *R* between matching and mismatching treatments for arbuscular mycorrhizal and ectomycorrhizal seedling types separately (*N* = 4 for each type) using Welch’s *t-*test (instead of a linear model and two-way ANOVA).

All statistical analyses were performed with R^48^, using the libraries vegan^49^ and lme4^50^.

## Electronic supplementary material

Supplementary Information

Description of Supplementary Data

Supplementary Data 1

Supplementary Data 2

## Data Availability

The fungal ITS sequences have been deposited in the databases under accession numbers BioProject PRJDB5467 and DDBJ DRA005499. All data analyzed during this study are included in this article (and its Supplementary Information files): seedling growth data used for calculating feedbacks (Supplementary Data 1) and fungal community dataset (Supplementary Data 2).
