# Supplementary material for: Mycorrhizal fungi mediate the direction and strength of plant–soil feedbacks differently between arbuscular mycorrhizal and ectomycorrhizal communities
Source: Commun Biol. 2018 Nov 20;1:196. doi: 10.1038/s42003-018-0201-9 (PMC6244237; doi:10.1038/s42003-018-0201-9)
Supplement: Supplementary file 1 — Supplementary Information [file 42003_2018_201_MOESM1_ESM.docx]

**Supplementary Figure 1**

**a b**

****  **
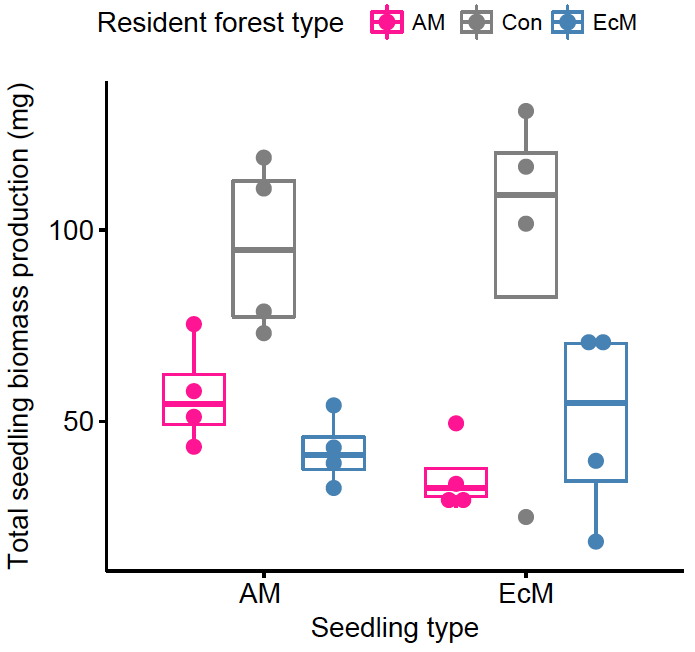
**

**c** AM seedling community **d** EcM seedling community

**
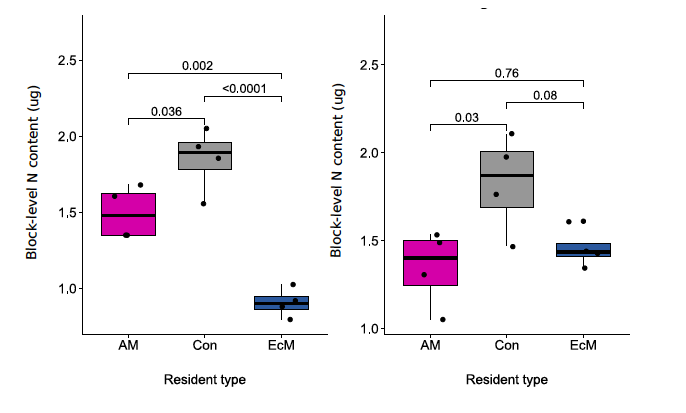
**

**Changes in soil chemistry, seedling community biomass and plant chemical contents**. (a) Principal component ordination of multivariate changes in the following soil characteristics: CEC, EC, pH-H_2_O, pH-KCl, NH_4_^+^, NO_3_^–^, P_2_O_5_, CaO, MgO, K_2_O, humus, Ca^2+^, Mg^+^, and K^+^. Different symbols indicate different treatment conditions indicated in the key (resident × seedling treatments). The shape of the symbols indicate resident types: rectangle, seedling control; circle AM (arbuscular mycorrhizal) resident treatment; triangle EcM (ectomycorrhizal) resident treatment. The colors of the symbols denote seedling treatments: pink, AM seedling treatment; gray, seedling control treatment, blue rectangle, EcM seedling treatment. (b) Boxplot of total seedling community biomass (mg) for replicate mesocosms (*i.e*., four seedling species combined) under different resident community types (each with different color; AM pink, control gray, and EcM blue). According to the results of general linear model, seedling type *P* = 0.299, Resident type *P* = 1.25e-3. Seedling × Resident *P* = 0.511. (c, d) Boxplots of leaf tissue N content under different resident types for arbuscular mycorrhizal seedling (AM) and ectomycorrhizal seedling (EcM) community types respectively. Each data point represents leaf N content averaged across all available seedling samples in a mesocosm for a subsample of seedling leaf tissue (192 seedlings). The means of the boxplots are statistically compared using Tukey’s multiple comparison testing.

**Supplementary Figure 2**

**Illustration of growth response calculation applied to our systems.** Symbols of different color represent different plant species.

**Supplementary Figure 3**

**Seedling growth responses under matching versus mismatching resident forest types based on leaf weight (the first row) and root weight (the second row).** Ln-transformed growth response *G_x_*(*y*) was calculated using leaf weight, and root weight for seven seedling species. A boxplot represents the minimum, the lower quartile, the median, the upper quartile and the maximum. *N* = 4 per seedling-resident species pair unless data were missing due to mortality. The boxplots of AM seedling species *Celtis* were not used for statistical analysis (*a priori* contrasts) as this species occurred only as seedling species, but not as resident species.

**Supplementary Figure 4**

**Taxonomic composition of the counts of the retrieved fungal operational taxonomic units (OTUs) identified from sapling species.** Pie charts are shown for each sapling species at the genus level based on the results of molecular analysis using high-throughput DNA sequencing. Numbers at the bottom left represent the total number of OTUs retrieved for the species. Putative functional groups are shown by different shades of red (AM fungi) and blue (EcM fungi).

**Supplementary Figure 5**

**a**

**b**

**Numbers of fungal OTUs associated with sapling and seedling species.** Based on the results of next-generation sequencing analysis of root-associated fungal communities, (a) number of OTUs present in roots of sapling species before the introduction of seedlings (left panel, before) and at the time of harvest (right panel, after), and (b) number of OTUs present in roots of seedling species upon the harvest, each panel grouped by different seedling species are shown.

**Supplementary Figure 6**


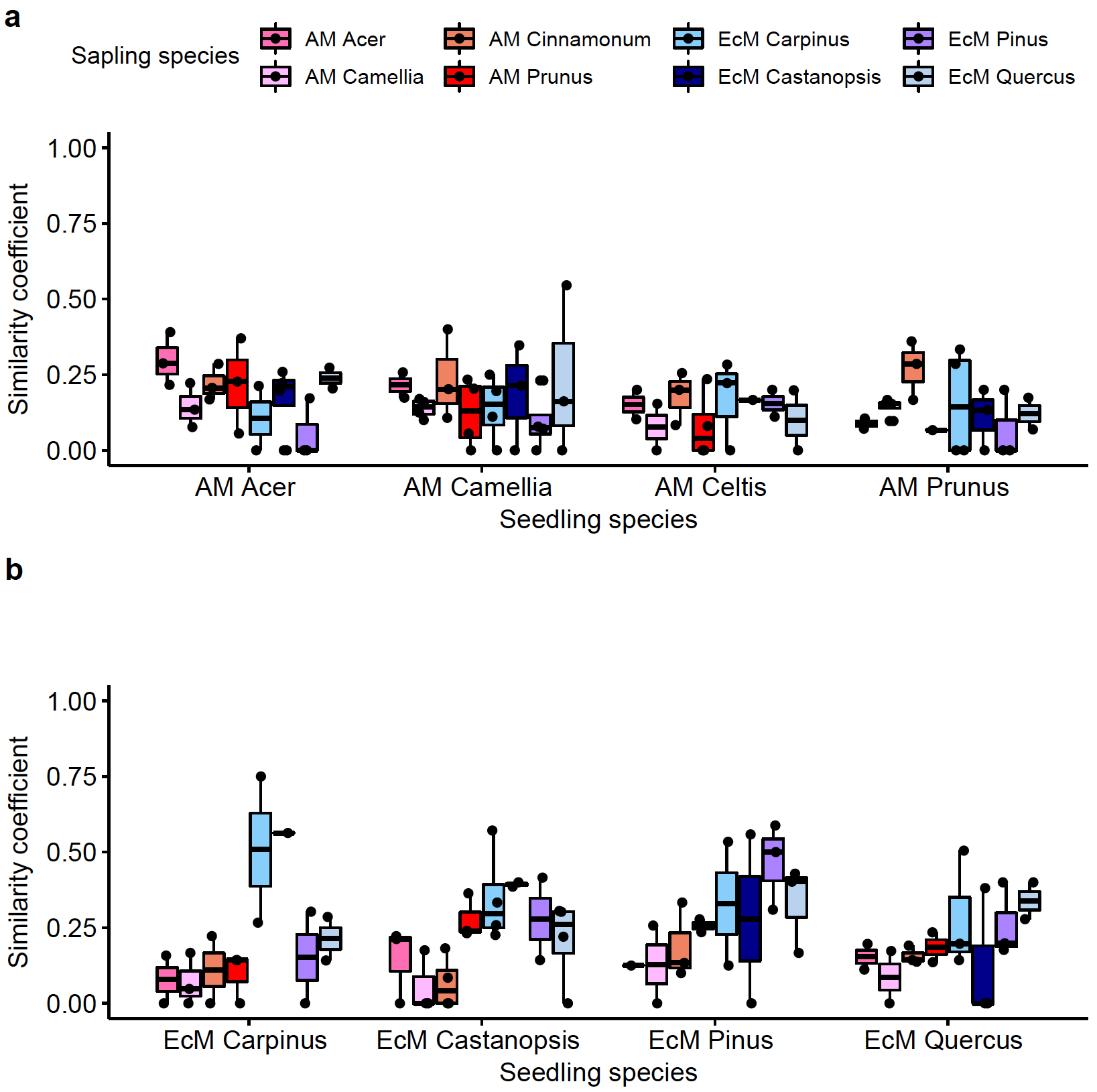


**Compositional similarities in fungal OTUs between neighboring sapling and seedling species.** Each dot represents a mesocosm-level mean similarity of all possible combinations of neighboring seedling and sapling species (*i.e.*, co-occurring in the same grids within a mesocosm). Each boxplot contains four data points (*i.e*., four block replicates) unless data were missing due to seedling mortality or other reasons. Similarity was quantified based on Morisita-Horn similarity coefficient.

**Supplementary** **Table 1**

**List of tree species**. AM, arbuscular mycorrhizal plant species; EcM, ectomycorrhizal plant species. For the arbuscular mycorrhizal seedling treatments, *Celtis* was used as a replacement of *Cinnamomum camphora*, because of the low germination rate.

| Species name | Family | Mycorrhiza | Life forms |
| --- | --- | --- | --- |
| *Castanopsis cuspidata* (Thunb.) Schottky | Fagaceae | EcM | Evergreen |
| *Carpinus laxiflora* (Sieb. et Zucc.) Blume | Betulaceae | EcM | Deciduous |
| *Pinus densiflora* Sieb. & Zucc. | Pinaceae | EcM | Evergreen |
| *Quercus serrata* Murray | Fagaceae | EcM | Deciduous |
| *Acer palmatum* Thunberg | Aceraceae | AM | Deciduous |
| *Camellia japonica* L. | Theaceae | AM | Evergreen |
| *Cinnamomum camphora* (L.) J. Presl | Lauraceae | AM | Evergreen |
| *Prunus* (*Cerasus) jamasakura* (Siebold ex Koidz.) H. Ohba | Rosaceae | AM | Deciduous |

**Supplementary Table 2**

**Results of linear mixed-effects model testing for sapling species effects on the seedling growth responses.** A linear mixed-effects model was fitted to single-species seedling growth response data. The estimated coefficient takes the form of *log* (*growth in heterospecific soil G_x_*(*y*) */growth in conspecific soil* *G_x_*(*x*)) and its deviation from zero (*i.e*., signature of positive or negative feedbacks) can be tested using *t*-test. AM, arbuscular mycorrhizal plant species; EcM, ectomycorrhizal plant species.

| Seedling species | Factor | Coefficient | SE | d.f. | *t* | *P* |
| --- | --- | --- | --- | --- | --- | --- |
| *Acer* | Intercept | -0.936 | 0.253 | 24.968 | -3.704 | **0.001** |
|  | AM *Camellia* | 0.556 | 0.355 | 21.758 | 1.566 | 0.132 |
|  | AM *Cinnamonum* | 0.271 | 0.355 | 21.758 | 0.762 | 0.454 |
|  | AM *Prunus* | 0.246 | 0.355 | 21.758 | 0.692 | 0.496 |
|  | Control | 0.748 | 0.355 | 21.758 | 2.105 | **0.047** |
|  | EcM *Carpinus* | 0.408 | 0.355 | 21.758 | 1.149 | 0.263 |
|  | EcM *Castanopsis* | -0.443 | 0.355 | 21.758 | -1.248 | 0.225 |
|  | EcM *Pinus* | -0.438 | 0.355 | 21.758 | -1.233 | 0.231 |
|  | EcM *Quercus* | -0.934 | 0.436 | 23.311 | -2.144 | **0.043** |
| *Camellia* | Intercept | 0.727 | 0.171 | 27.000 | 4.246 | **<0.001** |
|  | AM *Acer* | 0.123 | 0.242 | 27.000 | 0.509 | 0.615 |
|  | AM *Cinnamonum* | 0.236 | 0.242 | 27.000 | 0.973 | 0.339 |
|  | AM *Prunus* | 0.064 | 0.242 | 27.000 | 0.266 | 0.793 |
|  | Control | 0.304 | 0.242 | 27.000 | 1.255 | 0.220 |
|  | EcM *Carpinus* | 0.067 | 0.242 | 27.000 | 0.277 | 0.784 |
|  | EcM *Castanopsis* | 0.154 | 0.242 | 27.000 | 0.637 | 0.529 |
|  | EcM *Pinus* | 0.123 | 0.242 | 27.000 | 0.509 | 0.615 |
|  | EcM *Quercus* | 0.237 | 0.242 | 27.000 | 0.977 | 0.337 |
| *Prunus* | Intercept | -0.699 | 0.337 | 10.743 | -2.074 | 0.063 |
|  | AM *Acer* | 0.326 | 0.378 | 14.287 | 0.861 | 0.403 |
|  | AM *Camellia* | 0.201 | 0.348 | 14.105 | 0.577 | 0.573 |
|  | AM *Cinnamonum* | 0.527 | 0.348 | 14.105 | 1.514 | 0.152 |
|  | Control | 1.789 | 0.348 | 14.105 | 5.136 | **<0.001** |
|  | EcM *Carpinus* | -0.233 | 0.542 | 14.469 | -0.430 | 0.674 |
|  | EcM *Castanopsis* | -0.736 | 0.378 | 14.316 | -1.944 | 0.072 |
|  | EcM *Pinus* | -0.787 | 0.431 | 14.527 | -1.825 | 0.089 |
|  | EcM *Quercus* | -0.733 | 0.419 | 14.183 | -1.751 | 0.102 |
| Carpinus | Intercept | -1.107 | 0.513 | 24.379 | -2.160 | **0.041** |
|  | AM *Acer* | -0.920 | 0.690 | 23.019 | -1.333 | 0.196 |
|  | AM *Camellia* | -0.527 | 0.690 | 23.019 | -0.763 | 0.453 |
|  | AM *Cinnamonum* | -1.017 | 0.690 | 23.019 | -1.474 | 0.154 |
|  | AM *Prunus* | -0.342 | 0.690 | 23.019 | -0.495 | 0.626 |
|  | Control | 0.537 | 0.690 | 23.019 | 0.777 | 0.445 |
|  | EcM *Castanopsis* | -0.166 | 0.749 | 23.435 | -0.222 | 0.827 |
|  | EcM *Pinus* | -0.810 | 0.690 | 23.019 | -1.174 | 0.253 |
|  | EcM *Quercus* | -0.221 | 0.690 | 23.019 | -0.321 | 0.751 |
| *Castanopsis* | Intercept | -0.315 | 0.262 | 25.225 | -1.201 | 0.241 |
|  | AM *Acer* | -0.225 | 0.332 | 23.491 | -0.680 | 0.503 |
|  | AM *Camellia* | -0.385 | 0.332 | 23.491 | -1.160 | 0.258 |
|  | AM *Cinnamonum* | -0.540 | 0.332 | 23.491 | -1.627 | 0.117 |
|  | AM *Prunus* | -0.522 | 0.332 | 23.491 | -1.575 | 0.129 |
|  | Control | 0.445 | 0.332 | 23.491 | 1.340 | 0.193 |
|  | EcM *Carpinus* | 0.030 | 0.332 | 23.491 | 0.090 | 0.929 |
|  | EcM *Pinus* | -0.199 | 0.332 | 23.491 | -0.600 | 0.554 |
|  | EcM *Quercus* | -0.169 | 0.332 | 23.491 | -0.509 | 0.615 |
| *Pinus* | Intercept | 0.164 | 0.341 | 22.000 | 0.482 | 0.634 |
|  | AM *Acer* | -1.243 | 0.482 | 22.000 | -2.580 | **0.017** |
|  | AM *Camellia* | -1.889 | 0.482 | 22.000 | -3.920 | **0.001** |
|  | AM *Cinnamonum* | -1.194 | 0.451 | 22.000 | -2.649 | **0.015** |
|  | AM *Prunus* | -0.927 | 0.451 | 22.000 | -2.057 | 0.052 |
|  | Control | -0.061 | 0.451 | 22.000 | -0.135 | 0.894 |
|  | EcM *Carpinus* | 0.152 | 0.482 | 22.000 | 0.316 | 0.755 |
|  | EcM *Castanopsis* | -0.143 | 0.482 | 22.000 | -0.297 | 0.769 |
|  | EcM *Quercus* | -0.325 | 0.451 | 22.000 | -0.722 | 0.478 |
| *Quercus* | Intercept | 0.120 | 0.299 | 14.030 | 0.400 | 0.695 |
|  | AM *Acer* | 0.916 | 0.347 | 22.923 | 2.643 | **0.015** |
|  | AM *Camellia* | 0.490 | 0.347 | 22.923 | 1.413 | 0.171 |
|  | AM *Cinnamonum* | 0.517 | 0.377 | 23.087 | 1.371 | 0.183 |
|  | AM *Prunus* | 0.215 | 0.347 | 22.923 | 0.622 | 0.540 |
|  | Control | 1.023 | 0.347 | 22.923 | 2.952 | **0.007** |
|  | EcM *Carpinus* | 0.218 | 0.347 | 22.923 | 0.628 | 0.536 |
|  | EcM *Castanopsis* | 0.503 | 0.347 | 22.923 | 1.450 | 0.161 |
|  | EcM *Pinus* | 0.436 | 0.347 | 22.923 | 1.259 | 0.221 |

**Supplementary Table 3**

***A priori* contrasts testing for the effects of (a) neighbor sapling and (b) mycorrhizal type match/mismatch on the growth responses for seedling species.** (a) Comparing properties of conspecific versus heterospecific sapling neighbor of the matching mycorrhizal type. (b) Comparing for properties of the matching versus mismatching effects (comparing growth under matching versus mismatching heterospecific saplings). In each seedling species, we used *ln*-transformed growth response of seedling species *x*, that is, *ln*[*G_x_*(*y*)], as the response variable, sapling species identity *y* as a fixed-effects predictor, and block as a random-effects predictor: *ln*[*G_x_*(*y*)] ~ sapling species + (1|block). Bold *P*-values indicate statistical significance at the level of *α* = 0.05. AM, arbuscular mycorrhizal plant species; EcM, ectomycorrhizal plant species.

| (a) Properties of neighbor sapling | | | | |
| --- | --- | --- | --- | --- |
| Species | Coefficient | SE | *Z* | *P* |
| AM *Acer* | -1.294 | 0.524 | -2.471 | **0.014** |
| AM *Camellia* | 0.586 | 0.357 | 1.644 | 0.100 |
| AM *Prunus* | -1.050 | 0.588 | -1.787 | 0.074 |
| EcM *Carpinus* | -0.286 | 1.028 | -0.278 | 0.781 |
| EcM *Castanopsis* | 0.068 | 0.524 | 0.130 | 0.896 |
| EcM *Pinus* | 1.607 | 0.707 | 2.272 | **0.023** |
| EcM *Quercus* | -0.521 | 0.540 | -0.964 | 0.335 |
|  | | | | |
| (b) Properties of mycorrhizal type | | | | |
| Species | Coefficient | SE | *Z* | *P* |
| AM *Acer* | 0.710 | 0.202 | 3.514 | **4.42e-04** |
| AM *Camellia* | -0.004 | 0.131 | -0.033 | 0.974 |
| AM *Prunus* | 0.974 | 0.221 | 4.404 | **1.06e-05** |
| EcM *Carpinus* | 0.656 | 0.380 | 1.728 | 0.084 |
| EcM *Castanopsis* | 0.410 | 0.165 | 2.482 | **0.013** |
| EcM *Pinus* | 1.348 | 0.247 | 5.462 | **4.70e-08** |
| EcM *Quercus* | -0.096 | 0.194 | -0.495 | 0.620 |

**Supplementary Table 4**

**Number of seedling and sapling samples used for analyzing fungal communities.** Only the samples that passed the filtering procedure and were subject to statistical analysis are shown. AM, arbuscular mycorrhizal plant species; EcM, ectomycorrhizal plant species.

(a) Seedling species

| Species | Number of sample |
| --- | --- |
| AM *Acer* | 118 |
| AM *Camellia* | 128 |
| AM *Celtis* | 68 |
| AM *Prunus* | 60 |
| EcM *Carpinus* | 64 |
| EcM *Castanopsis* | 111 |
| EcM *Pinus* | 97 |
| EcM *Quercus* | 106 |

(b) Sapling species

| Species | Number of sample |
| --- | --- |
| AM *Acer* | 28 |
| AM *Camellia* | 29 |
| AM *Cinnamomum* | 30 |
| AM *Prunus* | 32 |
| EcM *Carpinus* | 36 |
| EcM *Castanopsis* | 29 |
| EcM *Pinus* | 34 |
| EcM *Quercus* | 31 |

**Supplementary Table 5**

**Treatment effects on changes in soil chemical composition**. The soil property data included 14 variables including CEC, EC, pH-H_2_O, pH-KCl, NH_4_^+^, NO_3_^–^, P_2_O_5_, CaO, MgO, K_2_O, humus, Ca^+^, Mg^+^, and K^+^. Before analysis, the absolute difference before seedling addition and after the experiment (at harvest) was calculated for each variable. Euclidean distance was used to represent multivariate dispersion.

| Variable | df | MS | *pseudo-F* | *R^2^* | *P* |
| --- | --- | --- | --- | --- | --- |
| Resident | 2 | 0.858 | 0.715 | 0.037 | 0.615 |
| Seedling | 2 | 1.495 | 1.245 | 0.064 | 0.276 |
| Resident × Seedling | 4 | 2.457 | 2.047 | 0.209 | **0.040*** |
| Residual | 27 |  |  | 0.690 |  |
| Total | 35 |  |  | 1.000 |  |

*****

Supplementary Note 1: Evidence for sapling-seedling competition

**Soil chemistry analysis.** The prerequisite for mycorrhizal fungi affecting seedling establishment is the presence of competition between saplings and seedlings for acquiring soil nutrients. In the absence of competition, there is no point for mycorrhizal fungi to compensate for or extend seedlings’ nutrient acquisition strategy. We evaluated if nutrient competition is reflected in changes in soil nutrient composition across treatment conditions during the two growing seasons in the experiment. We performed PERMANOVA using the soil nutrition dataset (Supplementary Methods: *Physiochemistry analysis*) to test for potential depletion of soil nutrients as a result of competition, that is, the net reduction of soil nutrients compared to a true control (*i.e.*, resident control × seedling control treatment). The formula of PERMANOVA (‘adonis’ function) was a two-way factorial design: *y* ≈ seedling community type + resident forest type + seedling community type: resident forest type, where *y* represents the standardized changes of the 14 soil component variables relative to the initial values at the beginning of the experiment. Significance testing was based on 9999 permutations. We used principal component analysis to visualize the results.

Changes in soil nutrient composition varied depending on treatment combinations (Supplementary Figure 1a; see Supplementary Table 5 for results; PERMANOVA, seedling × sapling interaction, *F* = 2.047, *P* = 0.040). To examine evidence for soil nutrient depletion as a potent mechanism affecting seedling growth, we analyzed soil P_2_O_5_ and found such a pattern: soil P_2_O_5_ was higher in the presence of ectomycorrhizal plant species and was lower in the presence of arbuscular mycorrhizal plant species compared to true control (*i.e.*, resident control ×seedling control treatment), irrespective of their sapling or seedling status. The most significant reduction (>120% reduction) was seen in the arbuscular mycorrhizal sapling × ectomycorrhizal seedling treatment compared to true control (GLM, *t* = –2.642, *P* = 0.013).

**Foliar nutrient analysis.** To examine if seedlings experience competition with saplings for acquiring soil nutrients, we compared leaf nitrogen content of eight seedling species across different resident forest treatments. If seedlings were confronted with competition under saplings, then one could expect seedlings to contain less nitrogen in leaves compared to the resident control treatments. To test this, we measured leaf nitrogen content, using a subset of dried foliar samples of surviving seedlings (192 seedlings; see Supplementary Methods: *Physiochemistry analysis*). Differences in seedling nitrogen were tested among different resident forest treatments for arbuscular mycorrhizal and ectomycorrhizal seedlings separately, using *ANOVA* and Tukey’s multiple comparison testing. The result showed that, for both arbuscular mycorrhizal and ectomycorrhizal seedlings, resident forest types had significant effects on the leaf-tissue nutrient content (arbuscular mycorrhizal seedling community, *F*_2,9_ = 32.85, *P =* 7.32e-05; ectomycorrhizal seedling community *F*_2,9_ = 5.54 *P* = 0.027). Generally, seedlings grown in the resident control treatments contained significantly higher nitrogen content in their leaves compared to the other resident treatments (Supplementary Figure1 c, d). These results confirmed reduced absorption of soil nitrogen by seedlings in the presence of the sapling community, supporting that competition for acquiring nutrients was a key driver of seedling growth responses in the experimental mesocosms.

Supplementary Note 2: Sequencing results

We harvested 1017 seedlings and 363 saplings for the downstream analysis. Prior to analysis, all dead seedlings were removed from the analysis. Over the course of the experiment, 353 of 1433 seedlings (24.6%) died. The numbers of dead seedlings per species were as follows: *Acer*, 52; *Camellia*, 8; *Celtis*, 58; *Prunus*, 27; *Carpinus*, 82; *Castanopsis*, 29; *Pinus*, 70; *Quercus*, 27. We used only a subset of the harvested samples because of the mortality events, sequencing quality assessment and the other bioinformatics data processing prior to statistical analysis (see Supplementary Methods, *Next-generation sequencing analysis of root-associated fungal communities*). The numbers of seedling and sapling samples used to produce the presence/absence matrices of fungal OTUs are summarized in Supplementary Table 4.

A total of 520 and 371 fungal OTUs were found in roots of seedling and sapling communities, respectively. The OTUs comprised mixtures of taxonomically distinct species (including 3 phyla, 86 families, and 126 genera). At the time of harvest, ectomycorrhizal saplings hosted almost exclusively ectomycorrhizal fungal OTUs, whereas arbuscular mycorrhizal saplings contained many arbuscular mycorrhizal fungal OTUs and an appreciable number of ectomycorrhizal fungal OTUs (Supplementary Figure 4).

Within the four ectomycorrhizal and four arbuscular mycorrhizal seedling species, 87.6% and 81.0% of OTUs occurred in the roots of more than two species of the same mycorrhizal type (excluding OTUs occurring only once), respectively. Low host plant species specificity indicates the potential for mycelial network formation involving compatible plant–fungal associations within mesocosms. Sequencing analysis confirmed that the saplings were colonized in their roots by diverse fungi from soil at the time of transplanting to mesocosms (Supplementary Figure 5a). Fungal infection of seedlings was observed upon harvest in resident control treatments (*i.e*., in the absence of initially mycorrhizal saplings; Supplementary Figure 5b), implying that spore deposition might have helped fungi colonize new seedlings. We thus verified that seedlings receiving inocula from saplings were colonized by fungal communities and that sapling fungal communities were still present at the end of the experiment.

**Supplementary Methods**

**Physiochemistry analysis.** To complement our understanding about sapling effects on seedling growth responses, we determined weight percent of leaf tissue C, N, and H using the combustion technique on a 2400 Series II CHNS/O Elemental Analyzer (PerkinElmer, Waltham, MA), for a collection of dried leaf samples (*N*= 192 seedling leaf samples chosen so as to give a balanced representation of all treatment combinations). The samples were oven-dried at 60°C for 3 days, pulverized in a 2 mL Eppendorf tube with two 4 mm zirconium balls for 3 min at 25 times s^-1^, and weighed to the nearest 0.001 mg.

To explore evidence for plant nutrient competition in mesocosm, we took soil samples at 5–10 cm depth at the nine grid intersection points in individual mesocosms at two time points (before the establishment of sapling communities, and at harvest of seedlings). We mixed all nice intersection samples to make a single soil sample to represent a mesocosm-level soil property at the time of collection. The soil sampling thus created 36 mesocosms × 2 time points (*i.e*., before and after), leading to a total of 72 mesocosm soil samples. The collected soil samples were air dried, sieved to remove coarse fragments and were then homogenized for each mesocosm. Soil samples were then standardized by weight, and the following soil chemical variables were analyzed by JAHT Co., Ltd. (Osaka, Japan): cation exchange capacity (CEC), electro-conductivity (EC), pH (H_2_O and KCl extracted), NH_4_^+^, NO_3_^–^, P_2_O_5_, CaO, MgO, K_2_O, humus, Ca^2+^, Mg^+^, and K^+^.

**Next-generation sequencing analysis of root-associated fungal communities.**  DNA extraction and PCR protocols are detailed elsewhere^1,2^. Briefly, five terminal roots (ca. 2 cm each) were subsampled from each sample, immersed in a single tube filled with 70% ethanol containing 1 mm zirconium balls, and then shaken for 2 min at 15 times s^-1^ using a TissueLyser II (Qiagen, Venlo, The Netherlands)^2^. Washed roots were frozen at −25°C and then pulverized by shaking at a rate of 20 times per sec for 3 min with 4 mm zirconium balls using a TissueLyser II.

Fungal DNA was extracted using a standard CTAB method. Fungal ITS2 regions were amplified by using a nested PCR method; the entire ITS region was first amplified and then the ITS2 region was amplified using fusion primers (for details see refs^1,2^). The PCR products were pooled and the PCR amplicons were purified using ExoSAP-IT (GE Healthcare, Little Chalfont, Buckinghamshire, UK) and a PCR purification kit (Qiagen). Sequencing was performed using 454 GS Junior (Roche Diagnostics, Indianapolis, IN, USA).

We performed OTU-picking and taxonomic assignment using Claident v0.2.2014.10.29^3^. First, low-quality 3′-tail positions (QV < 27) of each sequence were chopped, sequences longer than 400 bp were truncated to 400 bp, and sequences shorter than 200 bp were filtered out with the aid of the clfilterseq command of Claident. Then reads in each sample were de-replicated. The de-replicated sequences were subjected to the UCHIME v4.2.40 software to eliminate possible chimeras (“--minh 0.1 --mindiv 0.8” were given as command line arguments). The non-chimeric sequences were assembled with a minimum cutoff similarity of 97%, and consensus sequences, which were less likely to contain PCR and pyrosequencing errors than were original sequencing reads, were then constructed for respective samples (hereafter, within-sample consensus sequences). Assembling of the within-sample consensus sequences was then performed across samples with a minimum similarity setting of 97%. The resulting among-sample consensus sequences represented fungal OTUs. The among-sample consensus sequences were split at the head of the matched position of ACC CGC TGA ACT TAA GC (the LR0R primer) into ITS and LSU sequences.

To infer the taxonomy of respective ITS OTUs, we prepared a local BLAST database based on the “nt” database downloaded from the NCBI ftp server (http://www.ncbi.nlm.nih.gov/Ftp/) on 25 September 2014. The “nt” database was filtered by removing sequences without taxonomic information at the genus level. Based on the filtered “nt” database, taxonomic assignment of the OTUs was performed based on the Query-centric auto-k-nearest neighbor (QC-auto) method^3^, which gives the most accurate taxonomic identification results among the existing methods of automated DNA barcoding. The OTUs that were not identified as true fungi (kingdom Fungi) were not included in the following analysis.

The presence or absence of the fungal OTUs was recorded for each sample, and these data were arranged into a matrix in which the rows represented plant samples (either seedling or resident tree sample) and columns represented fungal OTUs (for raw data, see Supplementary Data 2). To compare fungal OTU diversity among samples on an equal sequencing coverage^4^, we first estimated the expected coverage values of a sample of size *m* for every sample (using “rareslope” function), and second applied rarefaction to the whole dataset at the coverage value 98% (at points where the slopes of individual sample rarefaction curves decrease to 0.02), using the “rrarefy” function in vegan^5^ of R^6^.

*Acknowledgements*

The fieldwork and lab experiments could not have been done with the help of the many students and staff, but here we name highly engaged students and staff, M Kodama, Y Kaneko, T Koizumi, A Narita, Y Sawanobori, Y Sugiyama, Y Unno, A Yumoto, P. X. Zhang, K Fukunaga.

**Supplementary References**

1. Toju, H., Yamamoto, S., Sato, H., Tanabe, A.S., Gilbert, G.S., & Kadowaki, K. Community composition of root-associated fungi in a *Quercus*-dominated temperate forest: “codominance” of mycorrhizal and root-endophytic fungi. *Ecology and Evolution*. DOI: 10.1002/ece3.546. (2013).
2. Toju, H., Sato, H., Tanabe, A. S. Diversity and spatial structure of belowground plant–fungal symbiosis in a mixed subtropical forest of ectomycorrhizal and arbuscular mycorrhizal plants. *PLoS ONE*, 9, e86566. (2014).
3. Tanabe, A. S. & Toju, H. Two new computational methods for universal DNA barcoding: A benchmark using barcode sequences of bacteria, archaea, animals, fungi, and land plants, *PLoS ONE*, 8, e76910. (2013)
4. Chao, A., & Jost L. Coverage-based rarefaction and extrapolation: standardizing samples by completeness rather than size. *Ecology*, 93, 2533−2547. (2012).
5. Oksanen, J. *et al*. vegan: community ecology package v.2.4-5 (R Foundation for Statistical Computing. (2018).
6. R Core Team. R: a language and environment for statistical computing (R Foundation for Statistical Computing, 2018) (2018).
