# Supplementary material for: Mycorrhizal fungi mediate the direction and strength of plant–soil feedbacks differently between arbuscular mycorrhizal and ectomycorrhizal communities
Source: Commun Biol. 2018 Nov 20;1:196. doi: 10.1038/s42003-018-0201-9 (PMC6244237; doi:10.1038/s42003-018-0201-9)
Supplement: Supplementary file 2 — Description of Supplementary Data [file 42003_2018_201_MOESM2_ESM.docx]

Description of Additional Supplementary Files

**File Name:** Supplementary Data 1

**Description:** Data of seedling growth responses, physiochemistry analysis of leaf and soil samples.

Spreadsheet1 (Seedling_Growth): Rows represent samples, and columns represent seedling identity code (including information about spatial block, location within mesocosm, location within grid), seedling heightRGR (relative growth rate based on apical stem height, mm), seedleafW (dry weight of leaf, mg), seedstemW (dry weight of stem, mg), seedrootW (dry weight of root, mg), survival (survivorship), seeddiam (stem diameter at base), seedname (seedling species name), resident (resident forest treatment or species name), Lint (light intensity), Block (spatial block).

Spreadsheet2 (CN_seedling): Rows represent samples, and columns represent CN_ID (sample identity code), Block (spatial block), Grid (location within mesocosm), Location (location within grid), Seedling_sp (seedling species name), Resident (neighboring sapling species within grid), C (carbon, μg), H(hydrogen, μg), and N (nitrogen, μg).

Spreadsheet3 (Soil_chemistry): Rows represent mesocosm-level samples, and columns represent Block (spatial block), No (identity of mesocosm within block), Date (date of sampling), CEC (Cation Exchange Capacity), EC (Electric conductivity), pH-H_2_O, pH-KCl, NH_4_ _N (ammonium), NO3_N (nitrate),　P_2_O_5_, CaO, MgO, K_2_O, Humus, Ca, Mg, and K. Units of the variables are μg unless indicated otherwise.

**File Name:** Supplementary Data 2

**Description:** Fungal community data and results of taxonomic identification

Spreadsheet1 (summary.matrix): The sample-by-OTU matrix. Rows represent samples (IP2671H01 seedling samples; IRBEFFE01 resident saplings at harvest; and IRE1TFY01 resident saplings at the start of the experiment). Columns represent fungal OUT identity, and the assigned taxonomic information is provided in spreadhseet2.

Spreadsheet2 (Claident_result): The results of taxonomic assignment using Claident. Rows represent identity of OTU, and columns represent query (identity of OTU), superkingdom, kingdom, subkingdom, phylum, class, subclass, order, suborder, family, subfamily, tribe, genus, and species.

Spreadsheet3 (Study_design): The study design of the experiment. This sheet provides information about Block (spatial block), Grid (location of grid), Resident (resident forest type), Res_species (sapling species), and Seedling (mycorrhizal type of seedling).
